# Nutrition and Aging: Nutritional Health Inequity 

**DOI:** 10.1155/2012/164106

**Published:** 2012-10-16

**Authors:** Joseph R. Sharkey, Julie Locher, Nadine Sahyoun, Sara Wilcox

**Affiliations:** ^1^Program for Research in Nutrition and Health Disparities, School of Rural Public Health, Texas A&M Health Science Center, College Station, TX 77843-1266, USA; ^2^Public Policy and Aging Program, University of Alabama at Birmingham, Birmingham, AL 35294-2041, USA; ^3^Department of Nutrition and Food Science, University of Maryland, College Park, MD 20742, USA; ^4^Department of Exercise Science and Prevention Research Center, Arnold School of Public Health, University of South Carolina, Columbia, SC 29208, USA

Good nutritional health is critically important for the prevention and management of nutrition-related health conditions as well as the prevention of cognitive and physical functional decline. The achievement and maintenance of good nutritional health is particularly challenging for the burgeoning and diverse older population. Many seniors experience nutritional health inequity as a result of gender, race or ethnicity, education or income, country of birth, disability, living arrangement, adequacy of social support, or geographic location. 

The five research articles in this special issue used qualitative and quantitative approaches to extend the conversation about nutritional health inequity in vulnerable seniors. E. Edfors and A. Westergren used semistructured interviews of homeliving older adults in Sweden to understand self-determination and individual approaches to acquiring, preparing, and consuming food. Using in-depth interviews, R. J. Green-LaPierre and colleagues in Nova Scotia focused on a vulnerable subset of seniors (lone women) and explored the experiences of how these women with limited resources access food. A. M. Albertson and colleagues used a US sample of older adults to examine the contribution of ready-to-eat breakfast cereals to overall diet quality. D. L. Huang and colleagues focused on adults with mobility disabilities and used in-depth interviews to highlight the special challenges they face in gaining access to and utilizing food resources. A. Westergen documented the use of action-oriented study Circles in Sweden to facilitate professional development and better patient outcomes in nursing homes. The papers highlight several subsets of seniors that face nutritional health disparities and suggest implications beyond the settings and subpopulations they study.

This special issue is representative of a growing movement among researchers as well as providers of service to focus on nutritional health equities among older adults who are most disadvantaged for a whole host of reasons. All of the authors brought attention to the many and varied challenges encountered in addressing nutritional health inequities. They all also emphasized the need for developing interventions targeted at addressing this persistent, yet addressable, social and health problem.

*Joseph R. Sharkey* *Joseph R. Sharkey* *Julie Locher* *Julie Locher* *Nadine Sahyoun* *Nadine Sahyoun* *Sara Wilcox* *Sara Wilcox*

